# A Novel Angular Acceleration Sensor Based on the Electromagnetic Induction Principle and Investigation of Its Calibration Tests

**DOI:** 10.3390/s130810370

**Published:** 2013-08-12

**Authors:** Hao Zhao, Hao Feng

**Affiliations:** 1 Department of Mechanical and Electrical Engineering, Jiaxing University, No.56, Yuexiu South Road, Jiaxing 314001, China; E-Mail: zhaohao@mail.zjxu.edu.cn; 2 Department of Information and Engineering Life, Hangzhou Dianzi University, No.1 Avenue, 2 Xiasha, Jianggan District, Hangzhou 310018, China

**Keywords:** sensor, angular acceleration, electromagnetic induction, mathematical model, calibration, error analysis

## Abstract

An angular acceleration sensor can be used for the dynamic analysis of human and joint motions. In this paper, an angular acceleration sensor with novel structure based on the principle of electromagnetic induction is designed. The method involves the construction of a constant magnetic field by the excitation windings of sensor, and the cup-shaped rotor that cut the magnetic field. The output windings of the sensor generate an electromotive force, which is directly proportional to the angular acceleration through the electromagnetic coupling when the rotor has rotational angular acceleration. The mechanical structure and the magnetic working circuit of the sensor are described. The output properties and the mathematical model including the transfer function and state-space model of the sensor are established. The asymptotical stability of the sensor when it is working is verified by the Lyapunov Theorem. An angular acceleration calibration device based on the torsional pendulum principle is designed. The method involves the coaxial connection of the angular acceleration sensor, torsion pendulum and a high-precision angle sensor, and then an initial external force is applied to the torsion pendulum to produce a periodic damping angle oscillation. The angular acceleration sensor and the angle sensor will generate two corresponding electrical signals. The sensitivity coefficient of the angular acceleration sensor can be obtained after processing these two-channel signals. The experiment results show that the sensitivity coefficient of the sensor is about 17.29 mv/Krad·s^2^. Finally, the errors existing in the practical applications of the sensor are discussed and the corresponding improvement measures are proposed to provide effective technical support for the practical promotion of the novel sensor.

## 1. Introduction

Angular acceleration is a common physical quantity in a rotating system, which can reflect the vibrational state of the rotation angle for the rotation shaft and transmission equipment while working. The responses of the rotation shaft and transmission equipment to various stimulations can be analyzed by measuring the angular acceleration, and especially the dynamic interference of rotary systems can be displayed in the form of angular acceleration. The measurement of rotating mechanical angular acceleration has wide applications, including the fields of automobiles, the military, aerospace, industry, electronics, *etc.* [1–5].

Angular acceleration can be measured indirectly by employing rotation angle sensors or angular speed sensors. However, the signal processing of these methods are very troublesome. Especially, the problems of delay characteristics and noise amplification are hard to resolve, which has prompted the development of direct angular acceleration measurement techniques. The applications of linear acceleration sensor are very wide [6–9], although it is more urgent to develop an angular acceleration sensor without the range limitation of the rotation angle. There exist the following problems in the presented research and development of angular acceleration sensors: lack of range limitation of the rotation angle, small phase lag, high signal-to-noise ratio and a wider band [10].

In recent years, some experts and scholars have conducted a series of researches on angular acceleration measurement. Moody [11] proposed a new sensitive angular accelerometer, which contained a superconducting test mass suspended by a weak flexure pivot; a superconducting quantum interference device amplifier and superconducting circuit were used to detect the angular displacement of the test mass. Sparks [12] described an electroformed micro-machining technology capable of producing both angular rate sensors and accelerometers, which were fabricated by CMOS and provided improved signal output. Jianli Li [13] proposed a novel micro electromechanical system (MEMS) pendulum angular accelerometer with electrostatic actuator feedback; it adopted a proof pendulum with optimized moment of inertia, suspended to dual anchors by a pair of torsion spring beams, as sensing component. Tomikawa [14] dealt with a piezoelectric angular acceleration sensor which can accurately distinguish a signal of rotational motion from other linear motion ones. In order to overcome the influence of gravity in angular acceleration measurements, a system for knee joint motions was constructed with a couple of accelerometers by Yamamoto [15]. The accurate angular acceleration could be calculated as the difference between two output values of the accelerometers. A bulk silicon micro-machined structure 6.4 mm × 6.4 mm in size designed and fabricated by Mizuno could detect angular acceleration and acceleration simultaneously and independently [16]. An angular acceleration measurement system without moving parts using a PVDF and a piezo-composite presented by Marat-Mendes had very low cost compared with other sensors [17]. A novel micro-fluidic channel angular accelerometer was proposed by Wolfaardt, and the sensor with a resolution of 15 mu rad/s^2^ and a bandwidth of 50 Hz was demonstrated [18].

In this paper, an angular acceleration sensor without the range limitation of the rotation angle is proposed to measure the instantaneous angular acceleration of the rotating system directly, based on the principle of electromagnetic induction. In addition, the installation of the sensor is very convenient, as the sensor just needs to be coaxially connected with the rotating system when it is used. The output signal of the sensor can be led directly without the slip ring to avoid the weakening of the signal. This paper describes the mechanical structure and working principle of this angular acceleration sensor, and constructs the mathematical model of the sensor, including the transfer function and state-space model. The asymptotical stability of the sensor is verified by the Lyapunov theorem when the sensor is working. Finally, the sensor calibration is demonstrated, and the probable existing errors and their impact on the applications of the sensor are analyzed.

## 2. The Structure and Principle of the Sensor

### 2.1. The Mechanical Structure of Sensor

**Figure 1.**
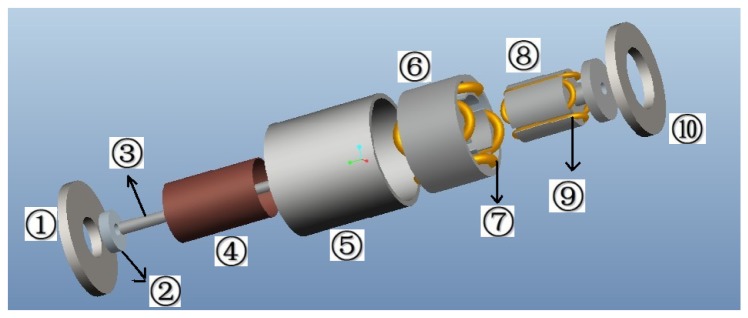
3D assembly diagram of the angular acceleration sensor.

**Figure 2.**
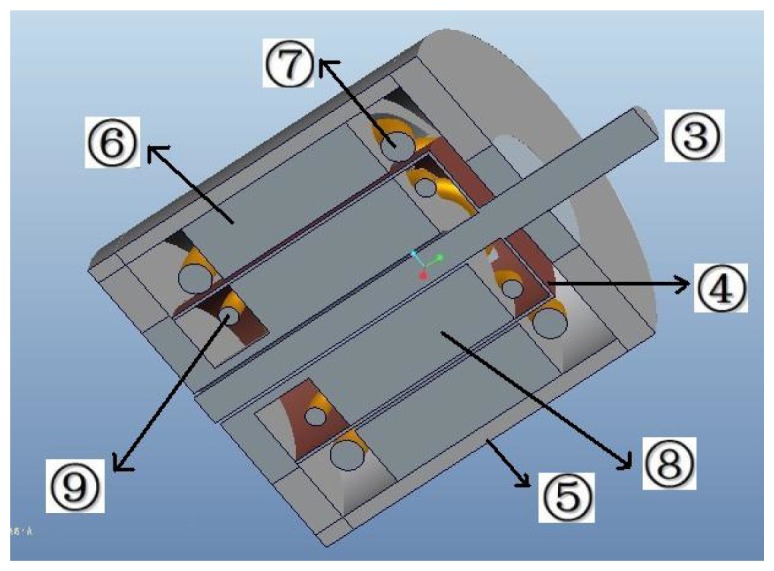
3D sectional view of the sensor.

**Figure 3.**
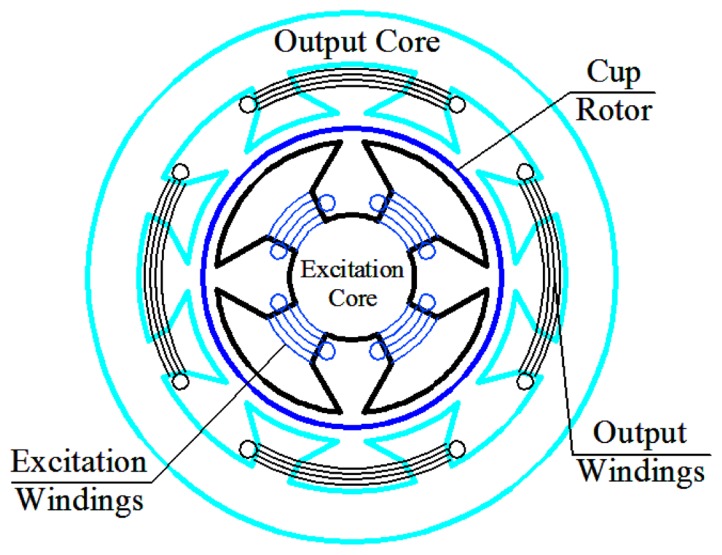
Sectional sketch view of the sensor.

The 3D assembly diagram of the angular acceleration sensor based on electromagnetic induction is shown in Figure 1, which includes front cover ➀, bearing ➁, sensor shaft ➂, cup-shaped rotor ➃, case ➄, outer stator core ➅, output windings ➆, excitation stator core ➇, excitation windings ➈, as well as the end cover ➉. The excitation stator core ➇ is composed of a high magnetic permeability soft magnetic iron-nickel alloy sheet or high magnetic permeability silicon steel through punching and shearing, and the excitation windings ➈ are embedded in its grooves; excitation windings ➈ are used for the generation of the constant air-gap magnetic field; the cup-shaped rotor ➃ is a thin-walled non-magnetic cup, which is prepared from high resistivity phosphor bronze, silicon manganese bronze and tin zinc bronze and other materials; the sensor shaft ➂ is connected coaxially with the shaft of the measured rotating system when measuring angular acceleration, and cutting the air-gap magnetic field formed by the excitation windings ➈; the outer stator core ➅ is made of the same materials as the excitation stator core ➇, and output windings ➆ are embedded in its grooves; the output windings ➆ will generate the electrical signal which corresponds to the angular acceleration of the rotating testing system. The corresponding 3D sectional view of the sensor is shown in Figure 2, and the sectional sketch map of the sensor is shown in Figure 3.

### 2.2. The Principle of Sensors

**Figure 4.**
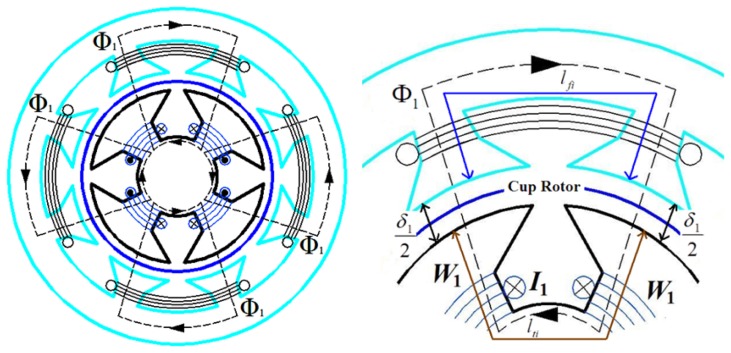
The magnetic circuit of Φ_1_.

The magnetic circuit is shown in Figure 4. When the excitation windings have DC current *I*_1_ assuming the magnetic circuit is symmetrical, the magnetic flux Φ_1_ of the magnetic circuit can be expressed by the following equation according to the magnetic circuit theorem [19]:

(1) $$\Phi_{1}=\frac{F_{1}}{R_{m1}}=\frac{2W_{1}I_{1}}{\frac{\delta_{1}}{\mu_{0}S_{1}}+\frac{l_{R}}{\mu_{R}S_{R1}}+\sum_{i=1}^{n}\left(\frac{l_{\text{fi}}}{\mu_{f}S_{\text{fi}}}+\frac{l_{\text{ti}}}{\mu_{t}S_{\text{ti}}}\right)}=K_{1}I_{1}$$

In which:
*W*_1_The effective windings number of the excitation windings*δ*_1_The effective length through the air-gap Φ_1_*S*_1_The effective area through the air-gap Φ_1_*μ*_0_The magnetic permeability of the air*l_R_*The effective thickness of the cup-shaped rotor*μ_R_*The magnetic permeability of the cup-shaped rotor*S*_*R*1_The effective area through the cup-shaped rotor Φ_1_*l_fi_*The each segment effective length through the outer stator core of magnetic flux Φ_1_*S_fi_*The each segment effective area through the outer stator core of magnetic flux Φ_1_*μ_f_*The magnetic permeability of the outer stator core*l_ti_*The each segment effective length through the inner stator core of magnetic flux Φ_1_*S_ti_*The each segment effective area through the inner stator core of magnetic flux Φ_1_*μ_i_*The magnetic permeability of the inner stator core
$K_{1}=\frac{2W_{1}}{\frac{\delta_{1}}{\mu_{0}S_{1}}+\frac{l_{R}}{\mu_{R}S_{R1}}+\sum_{i=1}^{n}\frac{l_{\text{fi}}}{\mu_{f}S_{\text{fi}}}+\frac{l_{\text{ti}}}{\mu_{t}S_{\text{ti}}}}$

The cup-shaped rotor can be seen as a number of squirrel-cage rotors. When the magnetic flux is cut counterclockwise by the cup-shaped rotor with a speed of *n* (unit is r/min), the generated motional electromotive force is:

(2) $$e_{R}=C_{e}\Phi_{1}n$$

where *C_e_* is the structure constant associated with the cup-shaped rotor, $C_{e}=\frac{\pi D}{60\tau }$, where *τ* is the pole moment of the stator and *D* is the outer diameter of the cup-shaped rotor. The resistance of the cup-shaped rotor is *r_R_*, and the reactance of cup-shaped rotor can be ignored, the current *i_R_* in the cup-shaped rotor is:

(3) $$i_{R}=\frac{e_{R}}{r_{R}}$$

**Figure 5.**
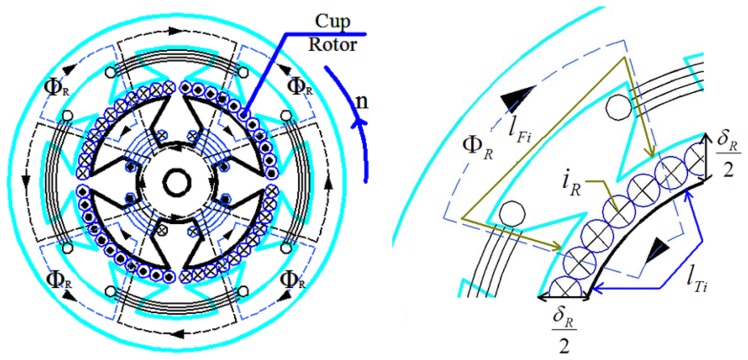
The magnetic circuit of Φ*_R_*.

*i_R_* generates a magnetic field, and its magnetic circuit is shown in Figure 5.

Similarly, the magnetic flux Φ*_R_* generated by *i_R_* can be expressed by the following equation according to the magnetic circuit theorem:

(4) $$\Phi_{R}=\frac{W_{R}i_{R}}{\frac{\delta_{R}}{\mu_{0}S_{R}}+\frac{l_{R}}{\mu_{R}S_{R2}}+\sum_{i=1}^{n}\left(\frac{l_{\text{Fi}}}{\mu_{f}S_{\text{Fi}}}+\frac{l_{\text{Ti}}}{\mu_{t}S_{\text{Ti}}}\right)}=K_{R}i_{R}$$

In which:
*W_R_*The equivalent number of windings for the cup-shaped rotor*δ_R_*The effective length through the air-gap Φ*_R_**S_R_*The effective area through the air-gap Φ*_R_**S*_*R*2_The effective area through the cup-shaped rotor of the magnetic flux Φ*_R_**l_Fi_*The each segment effective length of magnetic flux Φ*_R_* through the outer stator core*S_Fi_*The each segment effective area of magnetic flux Φ*_R_* through the outer stator core*l_Ti_*The each segment effective length of magnetic flux Φ*_R_* through the stator core*S_Ti_*The each segment effective area of magnetic flux Φ*_R_* through the stator core
$K_{R}=\frac{W_{R}}{\frac{\delta_{R}}{\mu_{0}S_{R}}+\frac{l_{R}}{\mu_{R}S_{R2}}+\sum_{i=1}^{n}\frac{l_{\text{Fi}}}{\mu_{f}S_{\text{Fi}}}+\frac{l_{\text{Ti}}}{\mu_{t}S_{\text{Ti}}}}$

The total effective windings of the output windings are *W*_2_. According to the principle of electromagnetic induction, the induction electromotive force in the output windings induced by the magnetic flux Φ*_R_* is exhibited as the following equation:

(5) $$e_{o}=-W_{2}\frac{d(2\Phi_{R})}{\text{dt}}$$

By the combination of Equations (1–5), the following equation can be obtained:

(6) $$e_{o}=\frac{-2K_{1}K_{R}C_{e}W_{2}W_{R}I_{1}}{r_{R}}\frac{\text{dn}}{\text{dt}}$$

From the above equation, we can find that the induction electromotive force of output windings *e_o_* is proportional to the rotation angular acceleration of the cup-shaped rotor.

## 3. The Mathematical Model of Sensor

### 3.1. Transfer Function

**Figure 6.**
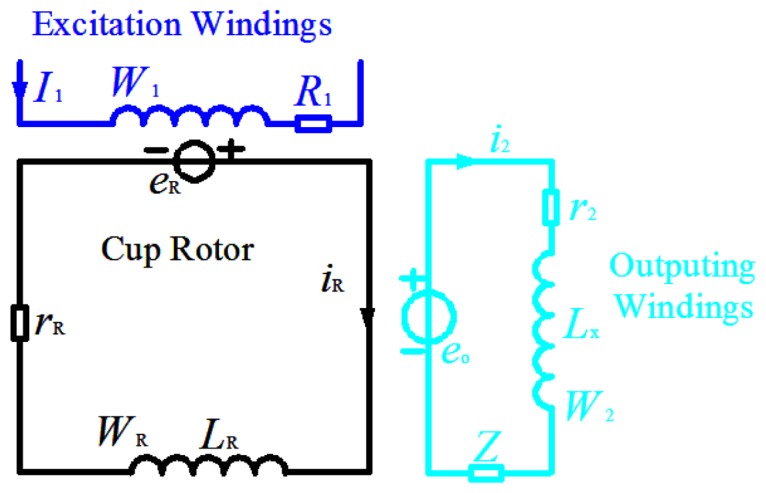
The equivalent circuit of the working sensor.

The equivalent circuit of the working sensor is shown in Figure 6:

In Figure 6:
*R*_1_The resistance of excitation windings*e_R_*The motional electromotive force of cup-shaped rotor*L_R_*The inductance of the cup-shaped rotor*r_R_*The resistance of cup-shaped rotor*i_R_*The current of cup-shaped rotor*e_o_*The induction electromotive force of output windings*i*_2_The current of output windings*L_x_*The leakage reactance of output windings*r*_2_The resistance of output windings*Z*The external load of output windings

For the cup-shaped rotor, the voltage balance equation is:

(7) $$e_{R}=i_{R}r_{R}+L_{R}\frac{di_{R}}{\text{dt}}$$

For the output windings, the voltage balance equation is:

(8) $$e_{o}=i_{2}r_{2}+L_{x}\frac{di_{2}}{\text{dt}}+i_{2}Z$$

The output voltage of sensor, namely the voltage across load is:

(9) $$u_{2}=i_{2}Z$$

The Laplace transform is demonstrated for the Equations (5–9), after combination, the transfer function of sensor is obtained:

(10) $$G(s)=\frac{L[u_{2}]}{L[\frac{\text{dn}}{\text{dt}}])}=\frac{u_{2(s)}}{\text{sn}(s)}=\frac{-C_{e}K_{1}K_{R}W_{2}ZI_{1}}{\left(r_{R}+L_{R}s)(r_{2}+Z+L_{x}s\right)}$$

It is seen that the intrinsic oscillation frequency (cutoff frequency) is $\omega_{n}=\sqrt{\frac{r_{R}(r_{2}+Z)}{L_{R}L_{x}}}$ and the damping ratio is $\xi =\frac{(r_{2}+Z)L_{R}+r_{R}L_{x}}{2\sqrt{(r_{2}+Z)L_{R}\cdot r_{R}L_{x}}}$ for the working sensor system, respectively. The poles of the system are $s_{1}=-\frac{r_{R}}{L_{R}}$ and $s_{2}=-\frac{r_{2}+Z}{L_{x}}$ respectively. Owing to the fact that the load resistance is large and the output windings leakage inductance *L_x_* is small, the dominant apex of the system is *s*_1_, that is $G(s)\approx -\frac{2C_{e}K_{1}K_{R}W_{2}ZI_{1}}{r_{R}+L_{R}s}$. The system can be approximated as a series of the proportional components and the inertial element, and the time constant of the inertial element is $\overset{L_{R}}{\;}/\underset{r_{R}}{\;}$. High resistivity material can be used to prepare the cup-shaped rotor, in order to improve the response speed of the system.

### 3.2. State-Space Model

Appropriate state variables are selected, the diagonal form of the system state-space model can be obtained through the transfer function of the system (10):

(11) $$\begin{array}{l} \left[\begin{array}{c} {ẋ}_{1}\\ {ẋ}_{2} \end{array}\right]=\left[\begin{array}{cc} -\frac{r_{R}}{L_{R}} & 0\\ 0 & -\frac{r_{2}+Z}{L_{x}} \end{array}\right]\;\left[\begin{array}{c} x_{1}\\ x_{2} \end{array}\right]+\left[\begin{array}{c} 1\\ 1 \end{array}\right]\frac{\text{dn}}{\text{dt}}=\text{A}\left[\begin{array}{c} x_{1}\\ x_{2} \end{array}\right]+\text{B}\frac{\text{dn}}{\text{dt}}\\ y=[\frac{2{\text{C}}_{e}K_{1}K_{R}W_{2}ZI_{1}L_{R}}{r_{R}L_{x}-(r_{2}+Z)L_{R}}\frac{2C_{e}K_{1}K_{R}W_{2}ZI_{1}L_{x}}{(r_{2}+Z)L_{R}-r_{R}L_{x}}]\left[\begin{array}{c} x_{1}\\ x_{2} \end{array}\right]=\text{C}\left[\begin{array}{c} x_{1}\\ x_{2} \end{array}\right] \end{array}$$

**Figure 7.**
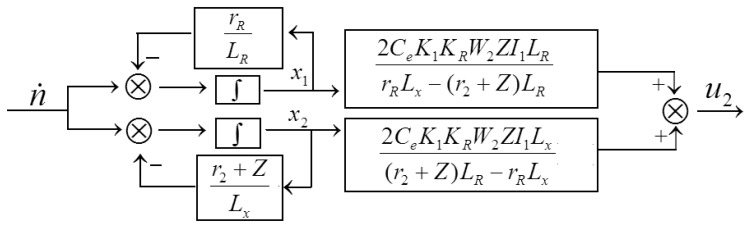
The state variables scheme of sensor.

The corresponding state variables scheme is shown in Figure 7:

The stability of the system can be divided into external stability and internal stability. The external stability is defined as the influence of the external input on the response of the system at the initial state, which can be judged according to the equation root situation of the closed-loop characteristic equation for the system. The internal stability is only related to the impact of the system itself at the initial state, also called Lyapunov asymptotical stability.

Assuming $\text{P}=\left[\begin{array}{cc} p_{1} & p_{2}\\ p_{3} & p_{4} \end{array}\right]$, the state matrix is introduced in Lyapunov equation, the solution is:

(12) $$\{\begin{array}{l} p_{1}=\frac{L_{R}}{2r_{R}}\\ p_{2}=p_{3}=0\\ p_{4}=\frac{L_{x}}{2(r_{2}+Z)} \end{array}$$

Owing to *p*_1_ > 0 and $\left|\begin{array}{cc} p_{1} & p_{2}\\ p_{3} & p_{4} \end{array}\right|=\frac{L_{R}L_{x}}{4(r_{2}+Z)r_{R}}>0$, the matrix *P* is positive definite, so the working sensor is Lyapunov asymptotic stable over a wide range.

## 4. The Sensor Calibration

### 4.1. Composition of the Calibration Equipment

**Figure 8.**
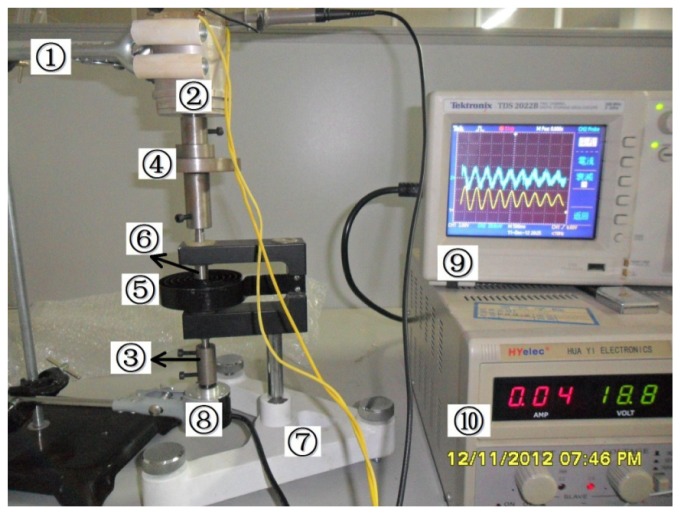
The calibration equipment of angular acceleration sensor.

The calibration equipment of the sensor is shown in Figure 8, which is composed of bracket ➀, angular acceleration sensor ➁, couplings ➂, mass ➃, coil spring ➄, torsion bar ➅, base ➆, angle sensor ➇, digital oscilloscope ➈, and DC power ➉.

The bracket is used to fix angular acceleration sensor ➁ and high-precision angle sensor ➇; The coupling ➂ is used for the coaxial connection of the angular acceleration sensor ➁ and high-precision angle sensor ➇; The mass ➃ is a self-made iron hoop with holes and has variable quality for selection and combination. The moment of inertia for the calibration system can be changed to realize calibration at different frequencies through selecting and combining masses; the combination of a large mass and a small mass is shown in the Figure; the coil spring ➄, the torsion bar ➅ and the base ➆ form a torsional pendulum, which is the power source of torsional vibration for the entire system. The torsion bar ➅ passes the mass and connects with the angular acceleration sensor ➁ through the upper end of the coupling ➃, and its bottom end is connected with the end of the angle sensor ➇; The dual channel input of the digital oscilloscope ➈ connects with the angular acceleration sensor and the angle sensor, respectively, for the acquisition and preservation of the output waveform of the two sensors during the experiments.

### 4.2. The Principle of the Calibration Equipment

An initial force is applied to the torsion bar ➅, resulting in the coil spring ➄ rotating an angle *θ* to produce the restoring moment *T*_1_*_s_* = *kθ*, in which *k* is the stiffness coefficient of the spring; The system begins to rotate under this restoring moment when the initial force disappears. In the rotation process, the system is affected by the air resistance torque $T_{2s}=-\gamma \frac{d\theta }{\text{dt}}$, in which *γ* is the drag torque coefficient; and also affected by the bearing friction resistance torque $T_{3s}=-\alpha \frac{d\theta }{\text{dt}}$, in which *α* is the friction torque coefficient; Assuming the system moment of inertia is *J*, we can obtain the following equation according to the torque balance equation:

(13) $$J\frac{d^{2}\theta }{dt^{2}}+\alpha \frac{d\theta }{\text{dt}}+\gamma \frac{d\theta }{\text{dt}}+k\theta =0$$

Assuming $2\beta =\frac{\gamma +\alpha }{J}$ and $\omega_{n}^{2}=\frac{k}{J}$, where *β* is the damping coefficient of the system, *ω_n_* is the intrinsic oscillation angular frequency of the system:

(14) $$\frac{d^{2}\theta }{dt^{2}}+2\beta \frac{d\theta }{\text{dt}}+\omega_{n}^{2}\theta =0$$

The solution for above equation is $\theta (t)=\frac{\theta_{0}}{\sqrt{1-\xi^{2}}}e^{-\beta t}\cos(\omega_{d}t-\varphi )$, in which *θ*_0_ is the initial amplitude of the pivot angle, $\omega_{d}=\omega_{n}\sqrt{1-\xi^{2}}$ is the damped oscillation angular frequency, damping ratio is $\xi =\frac{\gamma }{2\sqrt{\text{kJ}}}=\frac{\beta }{\omega_{n}}$. It is found that the vibration amplitude decays with time; the bigger the damping factor is, the faster it will decay. Assuming *T* is the cycle damping vibration for the pendulum, $\frac{\theta_{0}}{\theta_{N}}=e^{\beta \text{NT}}$ can be obtained if the initial amplitude *θ*_0_ and the amplitude *θ_N_* of the N-th cycle, as well as the used time of N cycles are measured. The damping coefficient can be expressed as:

(15) $$\beta =\frac{1}{\text{NT}}\ln\frac{\theta_{0}}{\theta_{N}}$$

The rotation cycle of the system is $T=\frac{2\pi }{\omega_{d}}=\frac{2\pi }{\omega_{n}\sqrt{1-\xi^{2}}}=\frac{2\pi }{\sqrt{\omega_{n}^{2}-\beta^{2}}}$, then $\frac{k}{J}=\frac{4\pi^{2}}{T^{2}}+\beta^{2}$ can be inferred. The angular rate of the pendulum is $\frac{d\theta }{\text{dt}}=0$ when system at the amplitude position. At this time, Equation (13) can be simplified as:

(16) $$\frac{d^{2}\theta }{dt^{2}}=-\frac{k}{J}\theta =-(\frac{4\pi^{2}}{T^{2}}+\beta^{2})\theta$$

where *β* is calculated by Equation (15) through experiments, and then the cycle *T* and the rotor angle *θ* are obtained through the output waveform of angle sensor saved by the oscilloscope. From Equation (16), we can get the actual angular acceleration value of the system, corresponding to the output voltage of the angular acceleration sensor. Additionally, we can also obtain the sensitivity coefficient of the angular acceleration sensor.

### 4.3. The Calibration Experiment Results

**Figure 9.**
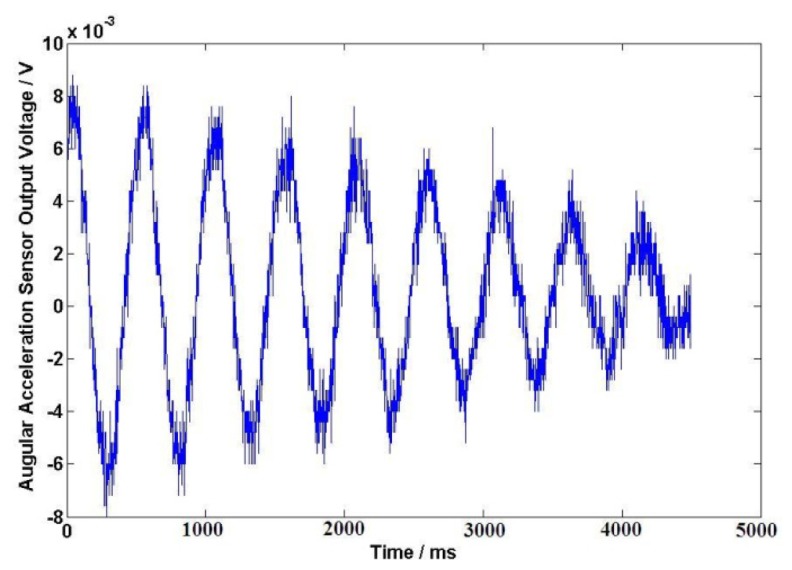
Output signals of angular acceleration sensor.

**Figure 10.**
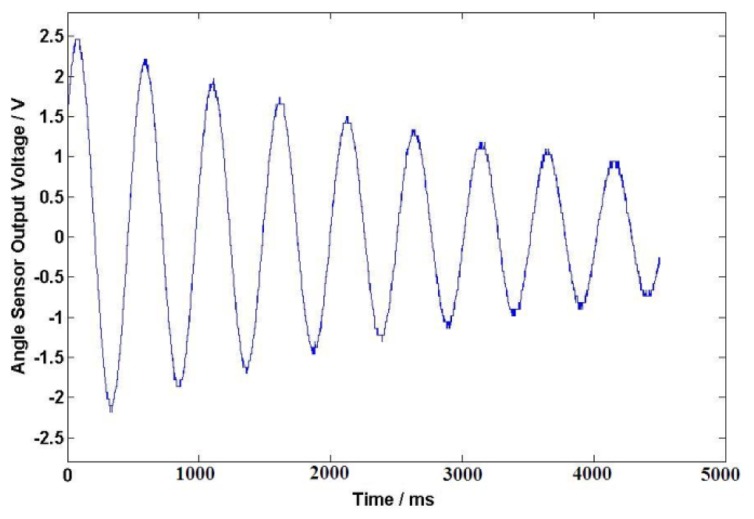
Output signals of angle sensor.

**Figure 11.**
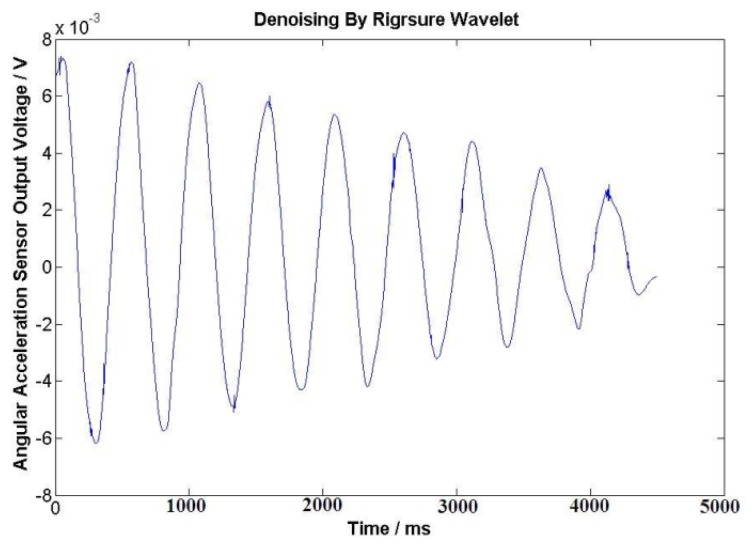
The de-noised signals by Rigrsure wavelet.

The DC resistance of the excitation windings for the angular acceleration sensor is 838 Ω. After a long-time test at the working temperature, excitation windings voltage *U*_1_ = 40 V is selected for the angular acceleration sensor. In this case, the temperature of the sensor is about 25 to 28 Celsius degrees; the initial angle is about 180° when the external force is applied on the coil spring, the output signals of angular acceleration sensor and the angle sensor are shown in Figure 9 and Figure 10 after the external force disappears. The output electrical signal of the angular acceleration sensor is de-noised by a Rigrsure wavelet; the obtained signal is shown in the Figure 11. The calculated results are shown in Table 1 by selecting the signal peaks of angular acceleration sensor and the angle sensor, according to the relevant formulas.

**Figure 12.**
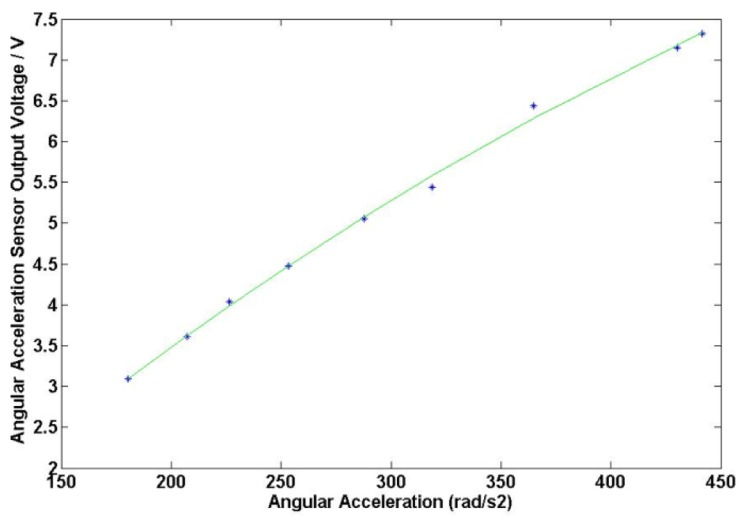
Calibration curve of angular acceleration sensor.

Matlab is used to fit the data in Table 1 and the obtained results are shown in Figure 12. It is found that the sensitivity coefficient of the angular acceleration sensor is about 17.29 mv/(Krad/s^2^).

### 4.4. Analysis of the Calibration Experiments

There will exist a phase difference when the angular acceleration sensor and the angle sensor are working in practice. From the calibration principle, we can see that the peak value of the angular acceleration is the corresponding amplitude position of the pendulum. Therefore, the peak voltages of the two sensors are needed in the calibration process and the phase difference shows little impact on the calibration results. Additionally, the aerostatic bearing can be considered to reduce the influence of bearing friction on calibration results; the calibration range of the system is limited and only suitable for the calibration of the angular acceleration in the lower frequency owing to the constraint of the mechanical structure and the limitation of variation range, even though the calibration frequency can be changed by the mass to a certain extent; the obtained calibration frequency is not continuous because the calibration system depends on the mass.

In order to solve the issues of non-continuous calibration point and lower frequency band, an auxiliary power source can be added to the electromagnetic force to realize a forced vibration state when the calibration equipment is working. The linear regulator of the vibration frequency can be realized by changing the rotational speed of the power source, resulting in the extension of the calibration frequency. These improvement ideas will be demonstrated in our future experiments.

## 5. Error Analysis

### 5.1. Fluctuations of Excitation Voltage

From the sensor principle, it is recognized that magnetic flux Φ_1_ generated by the excitation current includes the variation component ΔΦ_1_ if the excitation voltage has fluctuations. The cup-shaped rotor cuts the magnetic flux Φ_1_ with a constant rotation speed, so its motional electromotive force *e_R_* includes the variation component Δ*e_R_*, resulting in the existence of a current change component Δ*i_R_* for the cup-shaped rotor, and the generated magnetic flux Φ*_R_* also contains a variation component ΔΦ*_R_*. At this time, output windings will generate the corresponding induced electromotive force, known as the residual voltage. In order to overcome the abovementioned disadvantages of the sensor, a constant current source or a permanent magnet can be used to replace the existing excitation circuit to guarantee the constancy of the magnetic flux Φ_1_.

### 5.2. Effect of Temperature

**Figure 13.**
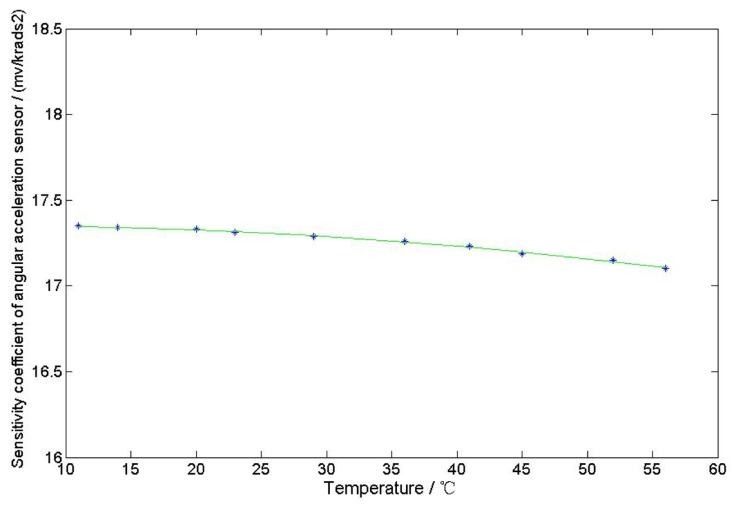
Temperature-sensitivity coefficient characteristics of the sensor.

The properties of the excitation windings, the output windings, the resistance of the cup-shaped rotor and the magnetic material may change because of the change in ambient temperature and the heating from extended use of the sensor. These property changes show influences on the output induced electromotive force of the sensor, resulting in the instability of the output performance. The character of the temperature-sensitivity coefficient of the sensor is shown in Figure 13. In this experiment, DS18B20 is used to detect the temperature of the sensor case. According to the results, the sensor sensitivity coefficient will decrease slightly with increasing temperature.

In order to decrease the impact of temperature, temperature compensation should be applied. A negative temperature coefficient thermistor can be concatenated in the excitation and the output circuit to compensate the temperature variation influence.

### 5.3. Nonlinearity Errors

The cup-shaped rotor of actual sensor is a first-order circuit composed of a resistance and inductance, so the change of the current *i_R_* lags induces an electromotive force *e_R_* due to the influence of the inductance *L_R_*, resulting in the distortion of the symmetrical magnetic circuit: the magnetic flux Φ*_R_* generated by the current *i_R_* varies with the change of rotational speed, and the change component of Φ*_R_* demagnetizes magnetic flux Φ_1_, causing the change of the magnetic flux Φ_1_. As a result, linear relationships of the output potential and the angular acceleration are destroyed, resulting in the presence of a non-linear error.

## 6. Conclusions

The angular acceleration sensor presented in this paper shows strong practicality without limitation on the rotation angle range. It only needs to connect with the measured rotating system coaxially when working. The structure and principle of the calibration equipment are described, and the calibration curves and performance indicators of the sensor are obtained by experiments. In our following work efforts will be made to examine the practical applications for this sensor in view of the extensive applications of angular acceleration sensors, such as transmission noise measurement and fault diagnosis for complex gearboxes or the internal combustion engine, fuel injection timing and fuel injection amount control for automotive engines, as well as the kinetic analysis for mechanical arm joints and so on.

## Figures and Tables

**Table 1.** Experimental calibration data.

The peak voltage of the angular acceleration sensor (mV)	7.32	7.15	6.44	5.44	5.06	4.48	4.04	3.61	3.09
The peak voltage of angle sensor (V)	2.30	2.24	1.90	1.66	1.50	1.32	1.18	1.08	0.94
The time corresponding peak (s)	0.071	0.586	1.098	1.606	2.12	2.626	3.138	3.656	4.158
Oscillation period T/(s)	(0.515 + 0.512 + 0.508 + 0.494 + 0.506 + 0.512 + 0.518 + 0.502)/8 = 0.5084								
Angle values (rad)	2.89	2.815	2.388	2.086	1.885	1.659	1.483	1.357	1.181
System damping ratio coefficient β	[ln(3.091÷1.181)] ÷8÷*T*≈0.2365								
Angular acceleration value (rad/s^2^)	−441.5	−430	−364.8	−318.6	−287.9	−253.4	−226.5	−207.3	−180.4
Sensitivity coefficient (mv/Krad/s^2^)	16.58	16.63	17.65	17.08	17.58	17.67	17.83	17.37	17.23
